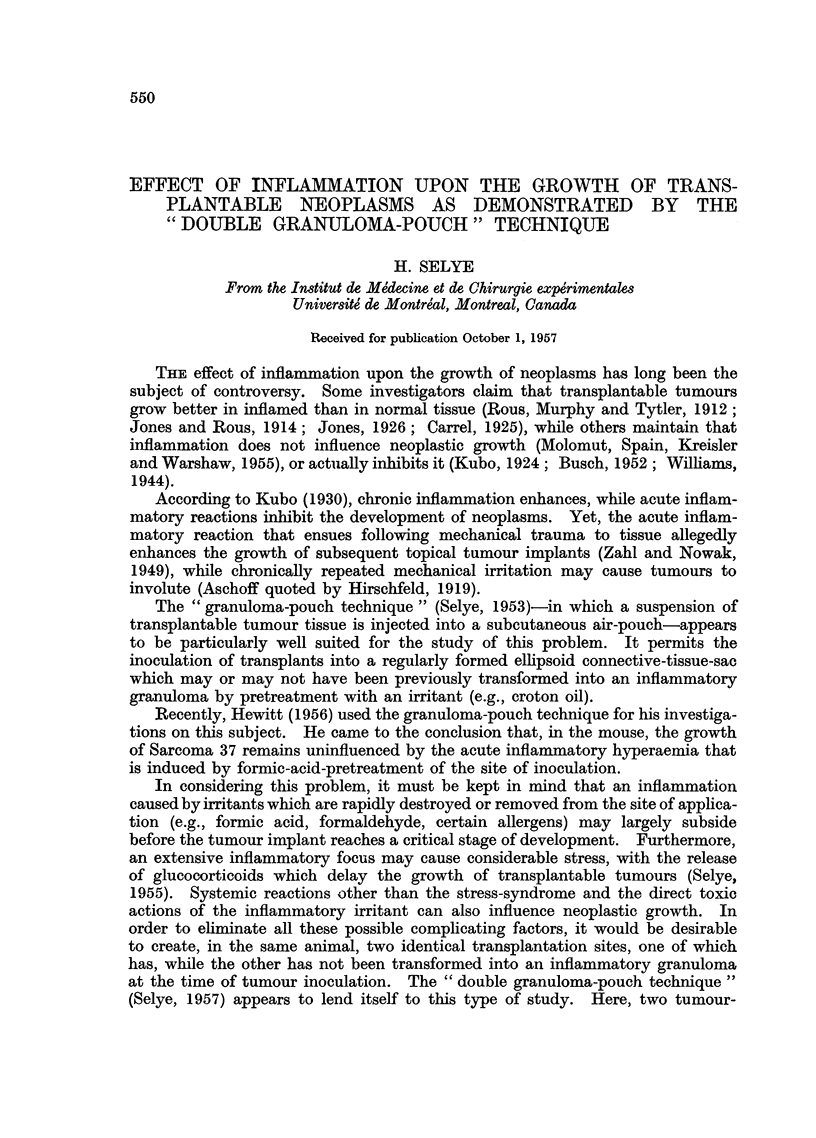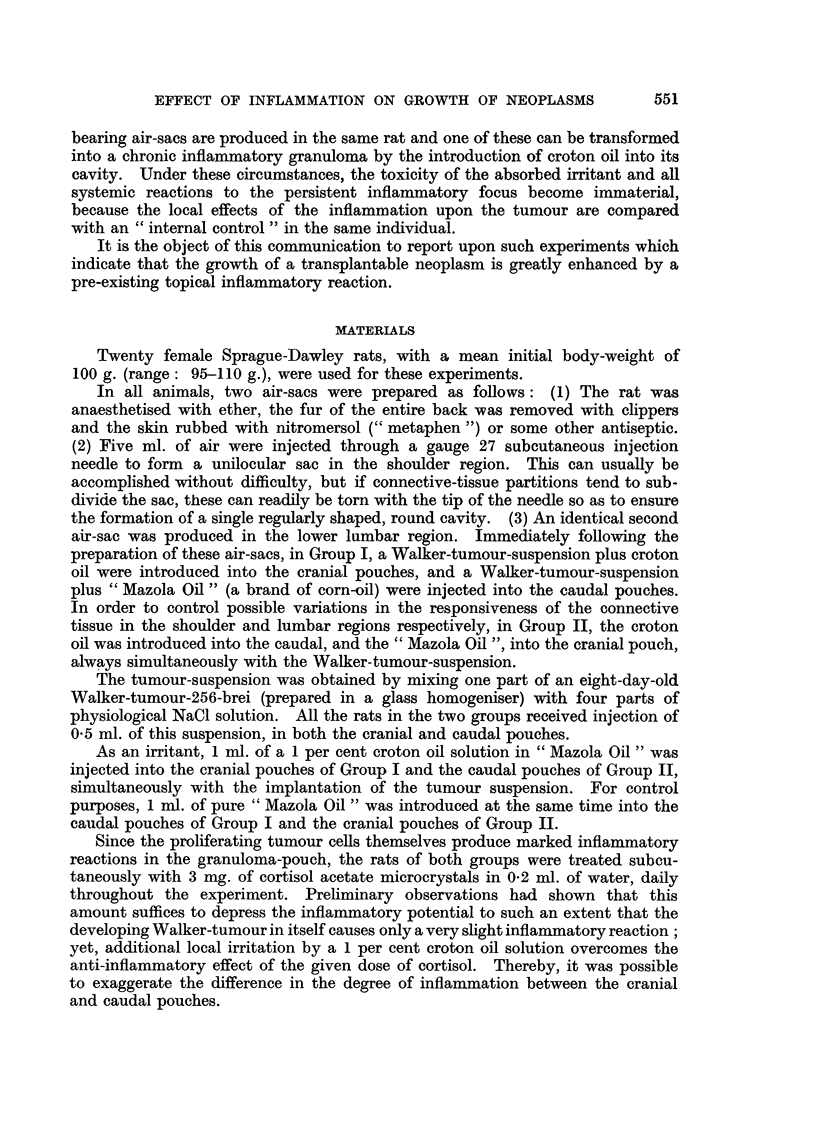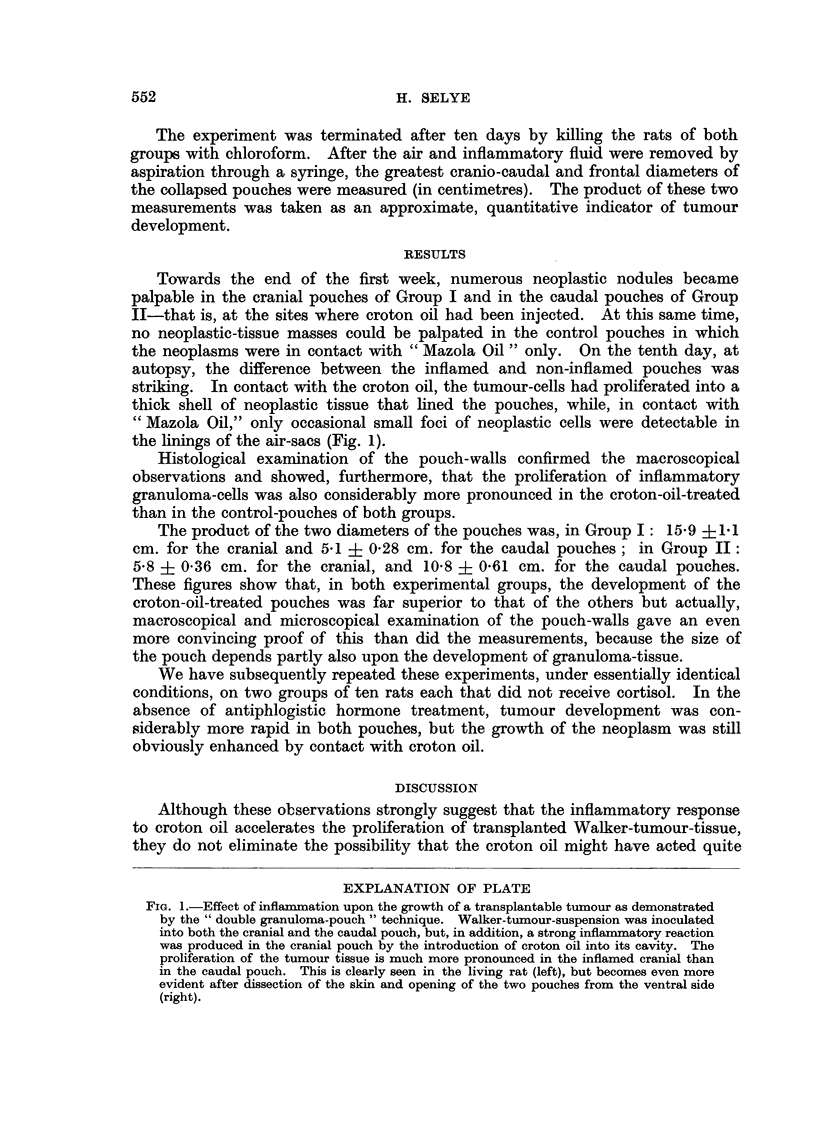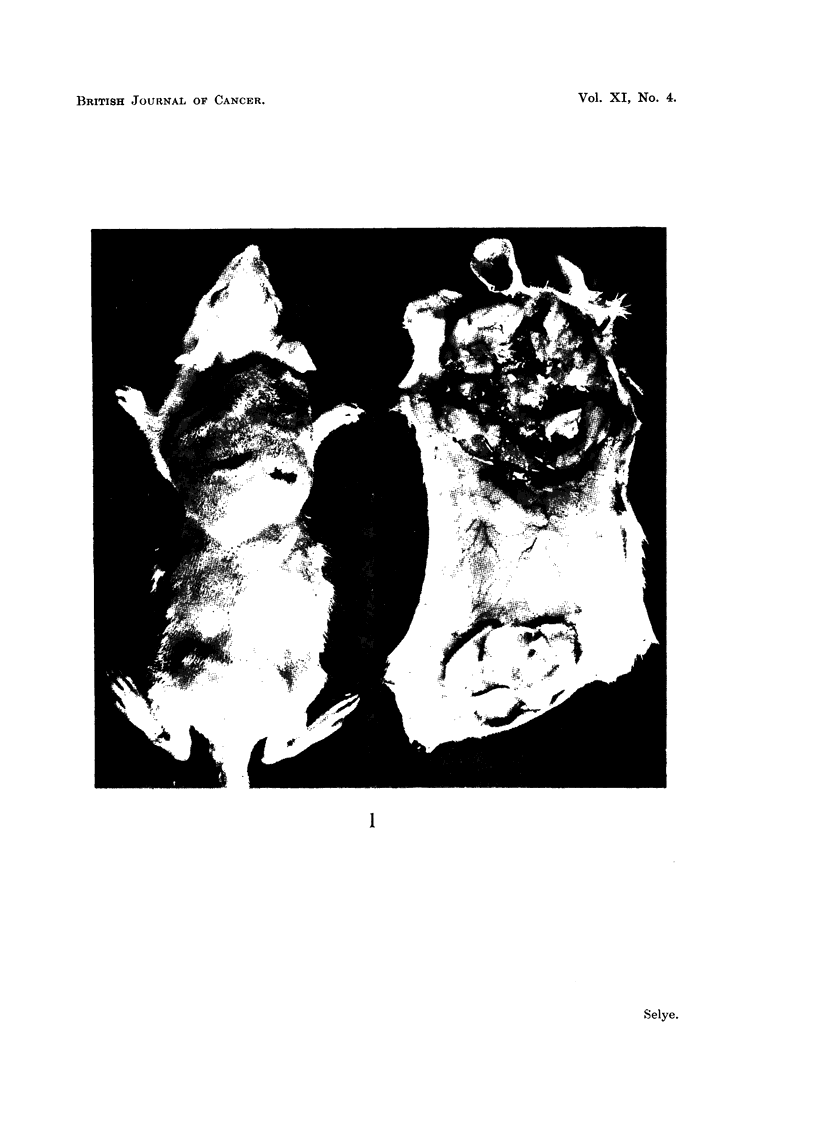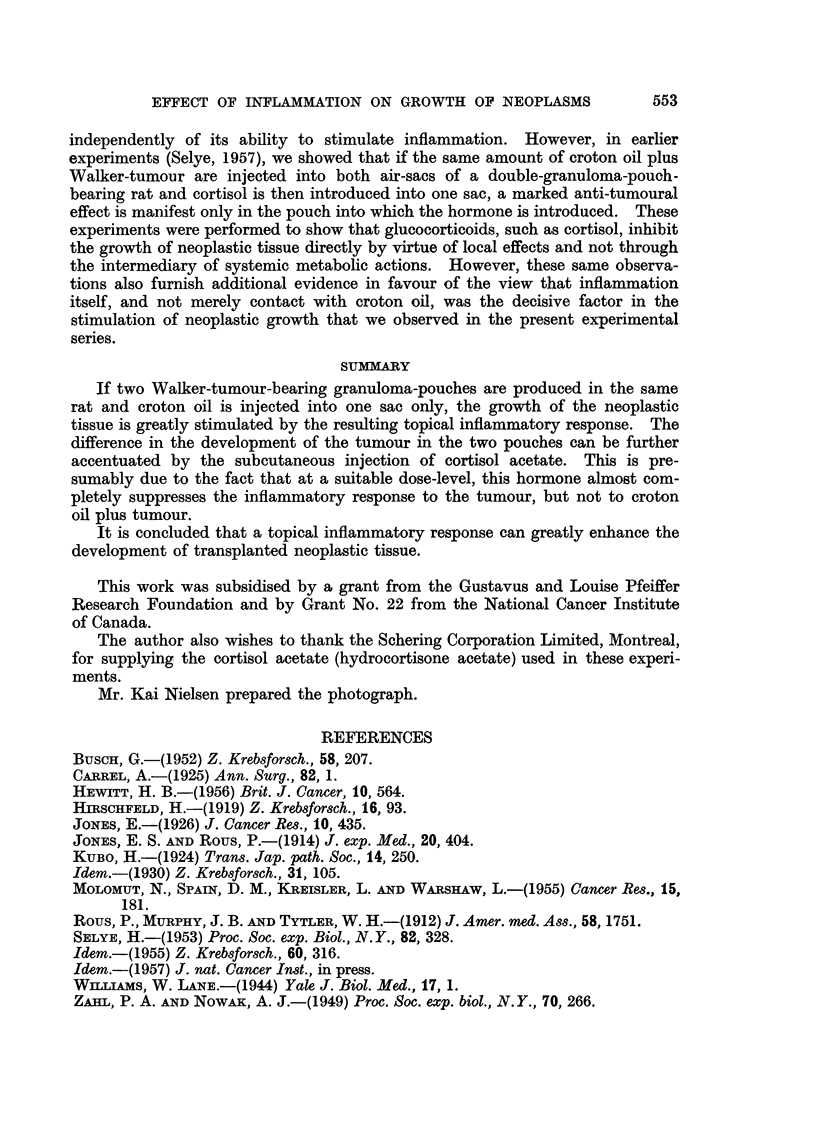# Effect of Inflammation upon the Growth of Transplantable Neoplasms as Demonstrated by the “Double Granuloma-Pouch” Technique

**DOI:** 10.1038/bjc.1957.67

**Published:** 1957-12

**Authors:** H. Selye

## Abstract

**Images:**


### EFFECT OF INFLAMMATION UPON THE GROWTH OF TRANSPLANTABLE NEOPLASMS AS DEMONSTRATED BY THE "DOUBLE GRANULOMA-POUCH" TECHNIQUE

H. SELYE

*From the Institut de Médecine et de Chirurgie expérimentales Université de Montréal, Montreal, Canada*

Received for publication October 1, 1957

THE effect of inflammation upon the growth of neoplasms has long been the subject of controversy. Some investigators claim that transplantable tumours grow better in inflamed than in normal tissue (Rous, Murphy and Tytler, 1912; Jones and Rous, 1914; Jones, 1926; Carrel, 1925), while others maintain that inflammation does not influence neoplastic growth (Molomut, Spain, Kreisler and Warshaw, 1955), or actually inhibits it (Kubo, 1924; Busch, 1952; Williams, 1944).

According to Kubo (1930), chronic inflammation enhances, while acute inflammatory reactions inhibit the development of neoplasms. Yet, the acute inflammatory reaction that ensues following mechanical trauma to tissue allegedly enhances the growth of subsequent topical tumour implants (Zahl and Nowak, 1949), while chronically repeated mechanical irritation may cause tumours to involute (Aschoff quoted by Hirschfeld, 1919).

The "granuloma-pouch technique" (Selye, 1953)—in which a suspension of transplantable tumour tissue is injected into a subcutaneous air-pouch—appears to be particularly well suited for the study of this problem. It permits the inoculation of transplants into a regularly formed ellipsoid connective-tissue-sac which may or may not have been previously transformed into an inflammatory granuloma by pretreatment with an irritant (e.g., croton oil).

Recently, Hewitt (1956) used the granuloma-pouch technique for his investigations on this subject. He came to the conclusion that, in the mouse, the growth of Sarcoma 37 remains uninfluenced by the acute inflammatory hyperaemia that is induced by formic-acid-pretreatment of the site of inoculation.

In considering this problem, it must be kept in mind that an inflammation caused by irritants which are rapidly destroyed or removed from the site of application (e.g., formic acid, formaldehyde, certain allergens) may largely subside before the tumour implant reaches a critical stage of development. Furthermore, an extensive inflammatory focus may cause considerable stress, with the release of glucocorticoids which delay the growth of transplantable tumours (Selye, 1955). Systemic reactions other than the stress-syndrome and the direct toxic actions of the inflammatory irritant can also influence neoplastic growth. In order to eliminate all these possible complicating factors, it would be desirable to create, in the same animal, two identical transplantation sites, one of which has, while the other has not been transformed into an inflammatory granuloma at the time of tumour inoculation. The "double granuloma-pouch technique" (Selye, 1957) appears to lend itself to this type of study. Here, two tumourbearing air-sacs are produced in the same rat and one of these can be transformed into a chronic inflammatory granuloma by the introduction of croton oil into its cavity. Under these circumstances, the toxicity of the absorbed irritant and all systemic reactions to the persistent inflammatory focus become immaterial, because the local effects of the inflammation upon the tumour are compared with an " internal control " in the same individual.

It is the object of this communication to report upon such experiments which indicate that the growth of a transplantable neoplasm is greatly enhanced by a pre-existing topical inflammatory reaction.

### MATERIALS

Twenty female Sprague-Dawley rats, with a mean initial body-weight of 100 g. (range : 95–110 g.), were used for these experiments.

In all animals, two air-sacs were prepared as follows : (1) The rat was anaesthetised with ether, the fur of the entire back was removed with clippers and the skin rubbed with nitromersol (" metaphen ") or some other antiseptic. (2) Five ml. of air were injected through a gauge 27 subcutaneous injection needle to form a unilocular sac in the shoulder region. This can usually be accomplished without difficulty, but if connective-tissue partitions tend to subdivide the sac, these can readily be torn with the tip of the needle so as to ensure the formation of a single regularly shaped, round cavity. (3) An identical second air-sac was produced in the lower lumbar region. Immediately following the preparation of these air-sacs, in Group I, a Walker-tumour-suspension plus croton oil were introduced into the cranial pouches, and a Walker-tumour-suspension plus " Mazola Oil " (a brand of corn-oil) were injected into the caudal pouches. In order to control possible variations in the responsiveness of the connective tissue in the shoulder and lumbar regions respectively, in Group II, the croton oil was introduced into the caudal, and the " Mazola Oil ", into the cranial pouch, always simultaneously with the Walker-tumour-suspension.

The tumour-suspension was obtained by mixing one part of an eight-day-old Walker-tumour-256-brei (prepared in a glass homogeniser) with four parts of physiological NaCl solution. All the rats in the two groups received injection of 0·5 ml. of this suspension, in both the cranial and caudal pouches.

As an irritant, 1 ml. of a 1 per cent croton oil solution in " Mazola Oil " was injected into the cranial pouches of Group I and the caudal pouches of Group II, simultaneously with the implantation of the tumour suspension. For control purposes, 1 ml. of pure " Mazola Oil " was introduced at the same time into the caudal pouches of Group I and the cranial pouches of Group II.

Since the proliferating tumour cells themselves produce marked inflammatory reactions in the granuloma-pouch, the rats of both groups were treated subcutaneously with 3 mg. of cortisol acetate microcrystals in 0·2 ml. of water, daily throughout the experiment. Preliminary observations had shown that this amount suffices to depress the inflammatory potential to such an extent that the developing Walker-tumour in itself causes only a very slight inflammatory reaction ; yet, additional local irritation by a 1 per cent croton oil solution overcomes the anti-inflammatory effect of the given dose of cortisol. Thereby, it was possible to exaggerate the difference in the degree of inflammation between the cranial and caudal pouches.

The experiment was terminated after ten days by killing the rats of both groups with chloroform. After the air and inflammatory fluid were removed by aspiration through a syringe, the greatest cranio-caudal and frontal diameters of the collapsed pouches were measured (in centimetres). The product of these two measurements was taken as an approximate, quantitative indicator of tumour development.

### RESULTS

Towards the end of the first week, numerous neoplastic nodules became palpable in the cranial pouches of Group I and in the caudal pouches of Group II—that is, at the sites where croton oil had been injected. At this same time, no neoplastic-tissue masses could be palpated in the control pouches in which the neoplasms were in contact with " Mazola Oil " only. On the tenth day, at autopsy, the difference between the inflamed and non-inflamed pouches was striking. In contact with the croton oil, the tumour-cells had proliferated into a thick shell of neoplastic tissue that lined the pouches, while, in contact with " Mazola Oil," only occasional small foci of neoplastic cells were detectable in the linings of the air-sacs (Fig. 1).

Histological examination of the pouch-walls confirmed the macroscopical observations and showed, furthermore, that the proliferation of inflammatory granuloma-cells was also considerably more pronounced in the croton-oil-treated than in the control-pouches of both groups.

The product of the two diameters of the pouches was, in Group I: $15{\cdot}9 \pm 1{\cdot}1$ cm. for the cranial and $5{\cdot}1 \pm 0{\cdot}28$ cm. for the caudal pouches; in Group II: $5{\cdot}8 \pm 0{\cdot}36$ cm. for the cranial, and $10{\cdot}8 \pm 0{\cdot}61$ cm. for the caudal pouches. These figures show that, in both experimental groups, the development of the croton-oil-treated pouches was far superior to that of the others but actually, macroscopical and microscopical examination of the pouch-walls gave an even more convincing proof of this than did the measurements, because the size of the pouch depends partly also upon the development of granuloma-tissue.

We have subsequently repeated these experiments, under essentially identical conditions, on two groups of ten rats each that did not receive cortisol. In the absence of antiphlogistic hormone treatment, tumour development was considerably more rapid in both pouches, but the growth of the neoplasm was still obviously enhanced by contact with croton oil.

### DISCUSSION

Although these observations strongly suggest that the inflammatory response to croton oil accelerates the proliferation of transplanted Walker-tumour-tissue, they do not eliminate the possibility that the croton oil might have acted quite

---

### EXPLANATION OF PLATE

FIG. 1.—Effect of inflammation upon the growth of a transplantable tumour as demonstrated by the " double granuloma-pouch " technique. Walker-tumour-suspension was inoculated into both the cranial and the caudal pouch, but, in addition, a strong inflammatory reaction was produced in the cranial pouch by the introduction of croton oil into its cavity. The proliferation of the tumour tissue is much more pronounced in the inflamed cranial than in the caudal pouch. This is clearly seen in the living rat (left), but becomes even more evident after dissection of the skin and opening of the two pouches from the ventral side (right).

1

independently of its ability to stimulate inflammation. However, in earlier experiments (Selye, 1957), we showed that if the same amount of croton oil plus Walker-tumour are injected into both air-sacs of a double-granuloma-pouch-bearing rat and cortisol is then introduced into one sac, a marked anti-tumoural effect is manifest only in the pouch into which the hormone is introduced. These experiments were performed to show that glucocorticoids, such as cortisol, inhibit the growth of neoplastic tissue directly by virtue of local effects and not through the intermediary of systemic metabolic actions. However, these same observations also furnish additional evidence in favour of the view that inflammation itself, and not merely contact with croton oil, was the decisive factor in the stimulation of neoplastic growth that we observed in the present experimental series.

SUMMARY

If two Walker-tumour-bearing granuloma-pouches are produced in the same rat and croton oil is injected into one sac only, the growth of the neoplastic tissue is greatly stimulated by the resulting topical inflammatory response. The difference in the development of the tumour in the two pouches can be further accentuated by the subcutaneous injection of cortisol acetate. This is presumably due to the fact that at a suitable dose-level, this hormone almost completely suppresses the inflammatory response to the tumour, but not to croton oil plus tumour.

It is concluded that a topical inflammatory response can greatly enhance the development of transplanted neoplastic tissue.

This work was subsidised by a grant from the Gustavus and Louise Pfeiffer Research Foundation and by Grant No. 22 from the National Cancer Institute of Canada.

The author also wishes to thank the Schering Corporation Limited, Montreal, for supplying the cortisol acetate (hydrocortisone acetate) used in these experiments.

Mr. Kai Nielsen prepared the photograph.

### REFERENCES

Busch, G.—(1952) *Z. Krebsforsch.*, **58**, 207.
Carrel, A.—(1925) *Ann. Surg.*, **82**, 1.
Hewitt, H. B.—(1956) *Brit. J. Cancer*, **10**, 564.
Hirschfeld, H.—(1919) *Z. Krebsforsch.*, **16**, 93.
Jones, E.—(1926) *J. Cancer Res.*, **10**, 435.
Jones, E. S. and Rous, P.—(1914) *J. exp. Med.*, **20**, 404.
Kubo, H.—(1924) *Trans. Jap. path. Soc.*, **14**, 250.
*Idem.*—(1930) *Z. Krebsforsch.*, **31**, 105.
Molomut, N., Spain, D. M., Kreisler, L. and Warshaw, L.—(1955) *Cancer Res.*, **15**, 181.
Rous, P., Murphy, J. B. and Tytler, W. H.—(1912) *J. Amer. med. Ass.*, **58**, 1751.
Selye, H.—(1953) *Proc. Soc. exp. Biol., N.Y.*, **82**, 328.
*Idem.*—(1955) *Z. Krebsforsch.*, **60**, 316.
*Idem.*—(1957) *J. nat. Cancer Inst.*, in press.
Williams, W. Lane.—(1944) *Yale J. Biol. Med.*, **17**, 1.
Zahl, P. A. and Nowak, A. J.—(1949) *Proc. Soc. exp. biol., N.Y.*, **70**, 266.